# Atrophic Pigmented Dermatofibrosarcoma Protuberans: A Case Report and Literature Review

**DOI:** 10.3389/fonc.2021.669754

**Published:** 2021-06-17

**Authors:** Juan Bai, Bin Liu, Taoming Liu, Jianjun Qiao, Hong Fang

**Affiliations:** ^1^ Dermatology Department, The First Affiliated Hospital, Zhejiang University School of Medicine, Hangzhou, China; ^2^ Department of Dermatology, Beilun Traditional Chinese Medicine Hospital, NingBo, China

**Keywords:** dermatofibrosarcoma protuberans, atrophic, pigmentation, sarcoma, bluish-black

## Abstract

Dermatofibrosarcoma protuberans (DFSP) is a rare soft tissue sarcoma characterized by the proliferation of spindle cells arranged in a storiform pattern. Here we report a case of DFSP presenting as a bluish-black atrophic plaque. The tumor had the histopathologic characteristics of both the pigmented and atrophic variants of DFSP. We describe the histopathologic, molecular, and imaging features of this patient and review other cases of DFSP that have been reported in the literature. Early identification of this DFSP variant can help clinicians implement clinical management strategies to prevent recurrence.

## Introduction

Dermatofibrosarcoma protuberans (DFSP) is a rare soft tissue sarcoma with an incidence of 0.8 to 4.5 cases per million persons per year (1). It is characterized by the proliferation of monomorphous spindle cells arranged in a distinctive storiform pattern. Classical DFSP often presents with a typical protuberant appearance. Its variants include fibrosarcomatous, myxoid, pigmented, giant cell, giant cell fibroblastoma, sclerotic, granular cell, and atrophic (2). Atypical clinical presentation and morphologic variants such as atrophic and pigmented DFSP may pose challenges for diagnosis (3–5). Six cases of DPSP with features of the pigmented and atrophic variants have been reported to date (3, 6–8). Here we report a new case of DFSP with features of pigmented and atrophic DFSP, and review the six published cases.

## Case Presentation

This study was approved by the ethics committee of The First Affiliated Hospital, Zhejiang University School of Medicine (approval no. IIT-2021-138). The patient signed a consent form for publication of case details and accompanying images.

**Figure 1:**
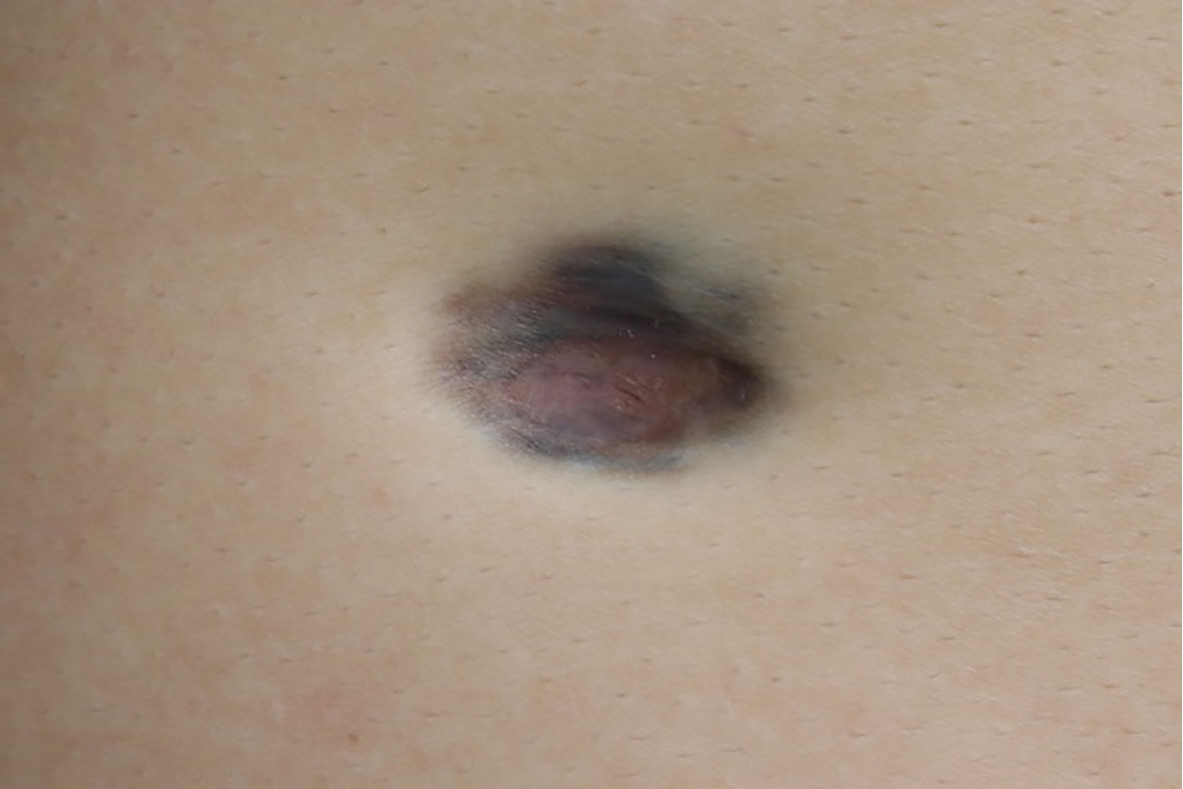
Bluish-black plaque with central atrophy on the left back of the patient.

**Figure 2:**
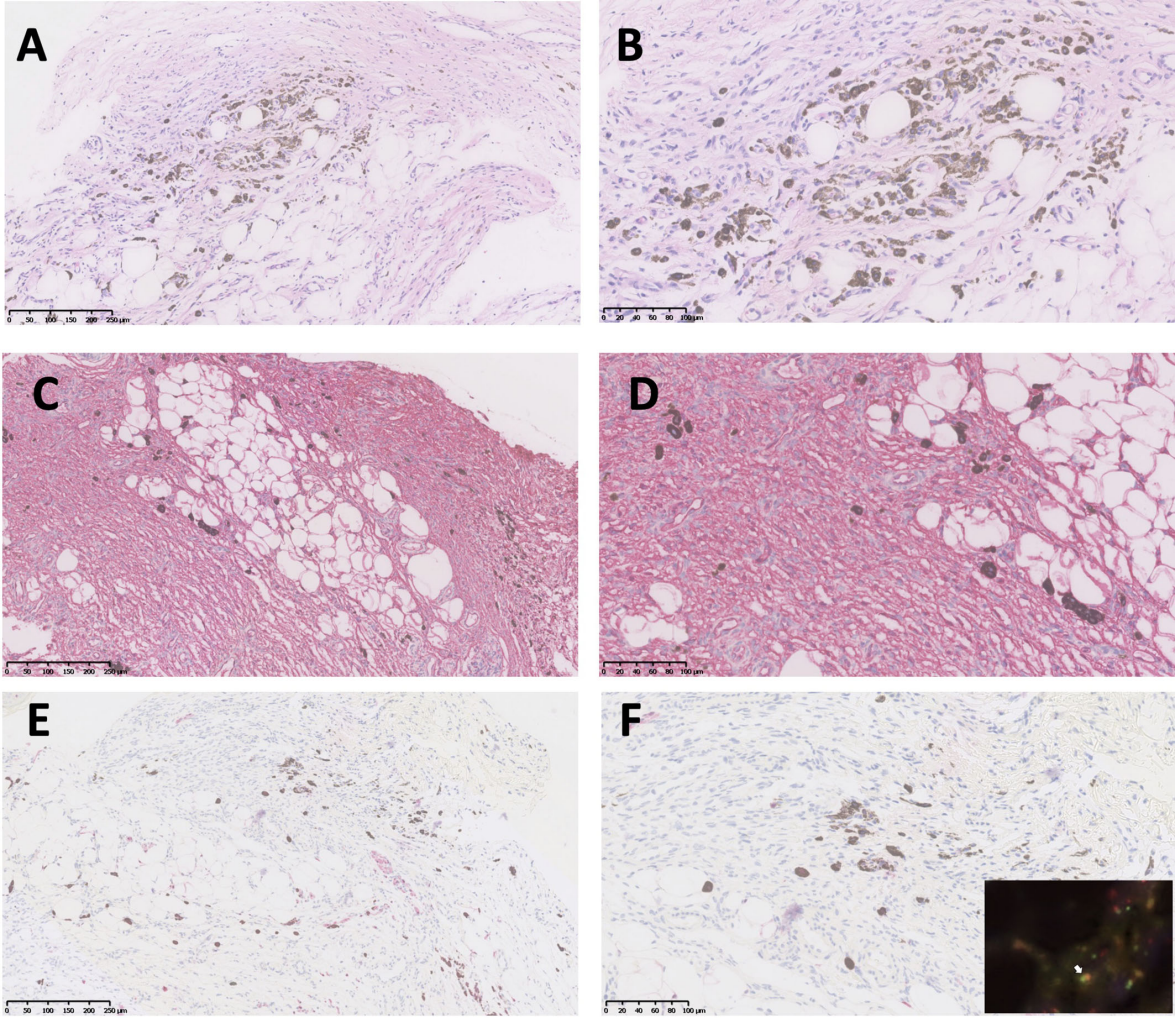
Histopathologic and immunohistochemical analyses of atrophic pigmented DFSP. **(A, B)** Proliferation of spindle cells in dermis and scattered pigmented cells (hematoxylin and eosin, 100× and 200× magnification). **(C, D)** Diffuse and strong expression of CD34 (100× and 200× magnification). **(E, F)** Pigmented cells positive for S100 (100× and 200× magnification). PDGFB and COL1A1 gene fusion was detected by FISH (**F**, insert, white arrow).

**Figure 3:**
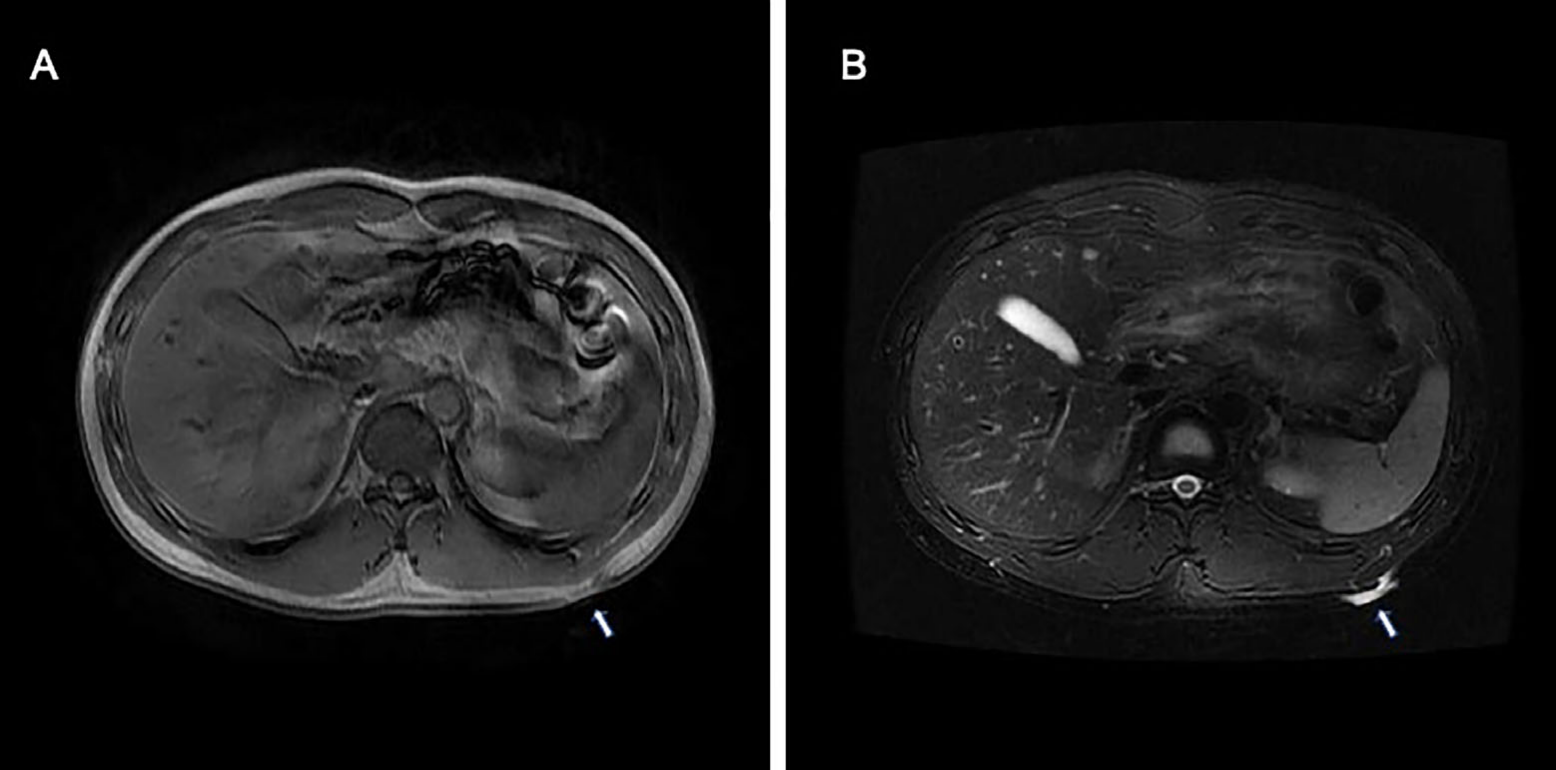
Magnetic resonance imaging of atrophic pigmented DFSP showed a well-circumscribed plaque with adjacent tissue thinning in the left region of the trunk. **(A)** T1-weighted images revealed a slightly higher non-uniform signal, **(B)** while T2-weighted images showed a high signal.

A 26-year-old man presented with a plaque on his left back that had been slowly progressing for 10 years. There was no history of trauma to the area. Physical examination showed a 30 × 25 mm asymptomatic, smooth, bluish-black plaque with central atrophy (**Figure 1**). Histopathologic analysis of the skin lesion biopsy revealed infiltration of spindle cells into the deep dermis and subcutaneous tissue, and the thickness of the regional dermis was <50% that of normal peripheral skin. Cells containing brown pigment were scattered in the dermis and subcutaneous tissue (**Figures 2A, B** **)**. Immunohistochemical analysis showed that the spindle cells were positive for cluster of differentiation CD34 (**Figures 2C, D** **)** and vimentin but negative for CD68, cytokeratin, desmin, and S100. The pigment-containing cells were positive for S100 (**Figures 2E, F** **)** but negative for CD34. The collagen, type I, alpha 1 (*COL1A1*)–platelet-derived growth factor subunit B (*PDGFB*) fusion gene was detected by fluorescence *in situ* hybridization (**Figure 2F**, insert). Magnetic resonance imaging showed a well-circumscribed plaque with adjacent tissue thinning in the left region of the trunk; T1-weighted images revealed a slightly higher non-uniform signal, while T2-weighted images showed a high signal (**Figures 3A, B** **)**. The patient was treated by wide local excision with 3-cm margins and reconstruction with a full-thickness skin graft. The patient has been regularly followed up for 7 months with no recurrence.

## Discussion

DFSP is a slow-growing soft tissue tumor with a high risk of local recurrence and low rate of distant metastasis (1). Characteristic cytogenetic findings of DFSP is chromosomal rearrangement of chromosomes 17 and 22, t (17,22) (q22; q13), which results in the fusion of the PDGFB to COL1A1, promoting continuous activation of PDGFR-beta in DFSP tumor cells. The *COL1A1*–*PDGFB* fusion gene should be analyzed in cases of unclear diagnosis or to identify candidates for tyrosine kinase inhibitor (TKI) treatment (1, 2, 4, 9).

The pigmented and atrophic variants of DFSP are rare, accounting for <5% (5) and 1.7% (3) of all cases, respectively. Pigmented DFSP, also known as Bednar tumor, often presents as a bluish-black discoloration similar to a bruise. Besides the typical histologic features of DFSP, these variants are characterized by the presence of melanin-containing dendritic cells (5, 10). The atrophic variant is easily misdiagnosed because of its variable presentation; it usually appears as a depressed plaque rather than protuberant nodules and manifests in the histologic examination as a dermis-based lesion showing a typical plaque-like growth pattern, with thinning of the dermis (3, 11).

Our patient had the features of both pigmented and atrophic DFSP in the same tumor, with evidence of two distinct cell populations in the immunohistologic analysis. Spindle cells were positive for vimentin and CD34, while pigmented dendritic cells were positive for S100.

Six cases of atrophic pigmented DFSP have been previously described (3, 6–8) (**Table 1**). The epidemiology of DFSP has no sex bias (1); the female:male ratio is 4:3 for atrophic pigmented DFSP, which is similar to the ratio of 2:1 for the atrophic variant (3). Trunk (3/7) and extremities (3/7) are the most frequently involved sites. DFSP typically occurs in young to middle-aged adults (1), whereas the atrophic pigmented form has also been observed in children.

**Table 1:** Clinical features of seven cases of atrophic pigmented dermatofibrosarcoma protuberans.

	Reference	Age, years	Sex	History, years	Location	Tumor morphology	Tumor size	Treatment
1	Chuan et al. (6)	24	Female	2	Left infraorbital	Well-demarcated bluish depressed area	NA	Untreated
2	Taura et al. (8)	34	Female	15	Left buttock	Pigmented plaque	11 × 12 mm	Wide excision
3	Zhang et al. (7)	7	Female	NA	Left wrist	Pigmented atrophic patch	20 × 40 mm	Wide excision
4	Zhang et al. (7)	8	Female	5	Left forearm	Bluish-back scleroatrophic plaque	10 × 10 mm	Wide excision
5	Xu et al. (3)	7	Male	4	Left forearm	NA	5mm	Wide excision
6	Xu et al. (3)	44	Male	3	Right back	Round bluish plaque-like lesion	25mm	Wide excision
7	This study	26	Male	10	Right back	Pigmented atrophic plaque	30 × 25 mm	Wide excision

NA, not available.

Due to histological overlap and CD34 immunostaining profiles, DFSP should be differentiated from other cutaneous spindle cell tumors including dermatofibroma, solitary fibrous tumor, neurofibroma, spindle cell or desmoplastic melanoma, intradermal spindle cell lipoma, and medallion-like dermal dendrocyte hamartoma (MLDDH). MLDDH is a rare congenital benign dermal lesion that shows clinical, histological, and immunohistochemical features that overlap with atrophic DFSP. Absent of the *COL1A1–PDGFB* fusion gene in MLDDH is the key identified point.

Treatment options for DFSP include wide local excision with negative margins and Mohs micrographic surgery (12). Radiotherapy and/or TKIs including imatinib (13) and sunitinib (14) may be used when surgical excision with clear margins is not feasible (1, 4, 15).

## Data Availability Statement

The original contributions presented in the study are included in the article/supplementary material. Further inquiries can be directed to the corresponding author.

## Ethics Statement

The studies involving human participants were reviewed and approved by the ethics committees of The First Affiliated Hospital, Zhejiang University School of Medicine. Written informed consent for participation was not required for this study in accordance with the national legislation and the institutional requirements. Written informed consent was obtained from the individual(s) for the publication of any potentially identifiable images or data included in this article.

## Author Contributions

JB managed the patient and drafted the manuscript. BL collected and analyzed the clinical data. TL reviewed literature and prepared **Table 1**. JQ and HF designed and revised the manuscript. All authors contributed to the article and approved the submitted version.

## Funding

This work was supported by grants from the National Natural Science Foundation of China (no. 81972931 to HF) and the Medical and Health Science and Technology Project of Health Commission of Zhejiang Province (no. 2020KY558 to JQ).

## Conflict of Interest

The authors declare that the research was conducted in the absence of any commercial or financial relationships that could be construed as a potential conflict of interest.
